# Evaluating Conservation Breeding Success for an Extinct-in-the-Wild Antelope

**DOI:** 10.1371/journal.pone.0166912

**Published:** 2016-12-09

**Authors:** Holly A. Little, Tania C. Gilbert, Marie L. Athorn, Andrew R. Marshall

**Affiliations:** 1 CIRCLE, Environment Department, University of York, York, North Yorkshire, United Kingdom; 2 Department of Animal and Plant Sciences, University of Sheffield, Sheffield, South Yorkshire, United Kingdom; 3 Marwell Wildlife, Winchester, Hampshire, United Kingdom; 4 Biology Department, University of York, York, North Yorkshire, United Kingdom; 5 Flamingo Land Ltd., Kirby Misperton, North Yorkshire, United Kingdom; University of Tasmania, AUSTRALIA

## Abstract

With the number of threatened species increasing globally, conservation breeding is vitally important now more than ever. However, no previous peer-reviewed study has attempted to determine how the varying conditions across zoos have influenced breeding by an extinct-in-the-wild species. We therefore use questionnaires and studbook data to evaluate the influence of husbandry practices and enclosure design on scimitar-horned oryx (*Oryx dammah*) breeding success, at the herd level. Regression models were used to identify the variables that best predicted breeding success among 29 zoos across a five-year period. Calf survival decreased with herd age and the use of soft substrates in hardstand areas (yard area usually adjacent to the indoor housing), explaining 30.7% of overall variation. Calf survival also decreased where herds were small and where food provisions were not raised (and hence likely incited competition), although these were less influential. Likewise, birth rate decreased with soft substrates in hardstand areas and unraised food provisions, although these were less influential than for calf survival. Birth rate increased with year-round male presence, yet this decreased calf survival. Compared to previous studies, the number of enclosure/husbandry influences on breeding were relatively few. Nevertheless, these few enclosure/husbandry influences explained over one third of the variation in calf survival. Our data therefore suggest some potential improvements and hence that extinct-in-the-wild species stand a greater chance of survival with empirical design of zoo enclosures and husbandry methods.

## Introduction

Anthropogenic influences are increasingly being shown to have a negative impact upon global biodiversity. Indeed, almost a quarter of extant mammals are currently classified as threatened and for certain species, this threat level has been exacerbated to a status of extinct-in-the-wild [1]. Nevertheless, conservation efforts have been proven to be successful. Of the 64 vertebrate species whose IUCN threat status has been reduced, zoological institutions are responsible for one-quarter of them [2]. Indeed, it has been estimated that with no conservation action (both *in situ* and *ex situ*), the IUCN status of ungulate species would be eight times worse than currently observed [3]. For mammals in particular, conservation breeding and reintroduction schemes have been more successful in improving conservation status than any other conservation action [2]. *Ex situ* conservation is thus a key tool in conserving threatened species, with managed captive populations acting as an insurance against extinction in the wild and in the case of species that are presently only found in captivity, is the only viable option [4].

However, conservation efforts are unlikely to have a significant impact on a species without unified actions, hence the IUCN SSC Conservation Breeding Specialist Group have recently launched a conservation framework titled the “One Plan Approach”. This framework outlines an integrated approach to conservation planning, in which all institutions and stakeholders associated with a particular species collaborate to develop a unified management plan [2]. Despite this, no previous study has attempted to determine how varying conditions across European zoos influence breeding in an extinct-in-the-wild species. Successful reintroduction programmes for these species depend exclusively upon *ex situ* population sustainability, which will be influenced by captive animal welfare [5]. Therefore, if conservation breeding programmes are to be successful for establishing wild populations, it would be beneficial to determine which particular aspects of enclosure design and husbandry promote optimal breeding success [6].

The most frequent feature of enclosure design found to influence zoo animal welfare and breeding is the overall land area (dholes [7]; giant pandas [8]; Humboldt penguins [9]; black rhinos [10]; southern hairy-nosed wombats [11]). Aside from space, additional influences on breeding success have included positive effects of mating groupings (red pandas [12]; striped skunks [13]), social structure (great apes [14]), feeding enrichment (elephants [15]) and colony size (Humboldt penguins [16]; chinstrap penguins [17]) and negative effects of dominance behaviour between males and females (black rhinos [18]), inter-zoo transfers (elephants [19]), calf separation (elephants [19]), age (elephants [15]; red wolves [20]; tigers [21]; European roe deer [22]; reindeer [23]; red-billed choughs [24]; western lowland gorillas [25]) and public exposure (black rhinos [10, 26]). However, few studies have addressed multiple variables of enclosure design and husbandry across multiple zoos [9], and are often biased by human perspective [6].

Scimitar-horned oryx (*Oryx dammah*), hereafter referred to as SHO, once inhabited the semi-arid steppe grasslands of North Africa [27], but were classified as being extinct-in-the-wild in 2000 [28] due to over-hunting, competition with domestic livestock and prolonged drought [29]. Nevertheless, SHO are a species with realistic prospects of recovery following trial reintroductions over the last 30 years [30] into Tunisia, Senegal, Morocco [29] and Chad [31]. Further successful conservation breeding is integral to maintaining a sustainable source population that can supply SHO for further releases into their natural habitat. SHO are also the second most commonly found antelope in managed populations [29], ensuring sufficient sample size unlike most previous assessments of animal welfare [9]. We therefore use SHO as a suitable case study for addressing how enclosure design can influence breeding success in extinct-in-the-wild species.

### Aim and Objectives

This study aims to show how enclosure design and husbandry measures can be used to evaluate conservation breeding success for extinct-in-the-wild species. We use SHO as a case study, through questionnaires and personal communication with animal managers. We quantify births, calf survivorship and enclosure and husbandry variables across institutions participating in the European Endangered species Programme (EEP). We use multivariate regression analysis to determine key enclosure design and husbandry features for breeding success at the herd level. Results are then used to provide recommendations for the future husbandry guidelines for SHO.

## Materials and Methods

### Questionnaire

Questionnaires on SHO enclosure design and husbandry variables thought to be important for SHO welfare [29] were sent to the 60 EEP institutions known to house SHO, with follow-up personal communication for clarification. Our questionnaires and the use of their data for this study were approved by the University of York Ethics Committee. Of these 60 institutions, we received completed questionnaires from 39 (65%), from which 29 (48%) were used in the study following exclusion of those including single sex groups or actively preventing breeding. The remaining 21 herds were largely non-breeding (51%) and hence our data are representative of the majority of breeding herds.

The questionnaires (S1 Appendix) consisted of 61 questions, which addressed (1) husbandry practices, including the use and type of environmental enrichment, human contact, the use of individual separation from the herd, male presence during parturition, public visibility of dams during parturition, diet variation, indoor/outdoor feeding method, transport and restraint methods, body condition and frequency and causes of injuries, (2) enclosure design, including cohabitation with other species, recent exhibit change, paddock, hardstand and stable size and stable and hardstand substrate types and (3) public influences, including minimum public distance to the SHO, proportion of the enclosure perimeter with public accessibility, barrier height and annual footfall.

Additional predictor variables (mean age of mature individuals, number of transfers, herd size and sex ratio) and breeding data were extracted from the SHO international studbook for all institutions participating in the study for the years 2010–2014. This period was chosen due to coinciding with an extensive plan by conservation biologists for a large-scale trial release of SHO into the wild and hence there being a clear need for a sustainable insurance population. Breeding data consisted of birth rate, 30-day calf survivorship and 24-month calf survivorship (Table 1).

**Table 1:** Summary values of SHO management and breeding success variables (for 2010–2014) included in the analyses.

Variable	Mean (95% CI) [and min-max]	Description	Rationale for inclusion
**Breeding success**			
Birth rate	● 1.4 (1.2–1.7) [0.0–2.7]	Number of births (including stillbirths) per female (Number of births/mean number of females in the herd)	A standard measure of breeding success [29]
30-day calf survivorship	● 0.7 (0.6–0.8)[0.0–1.0]	Proportion of calves born surviving to 30 days old i.e. deaths by 30 days (Number of calves that survived to 30 days/number of births)	A standard way of evaluating juvenile mortality, representing the month following parturition [29]
24-month calf survivorship	● 0.6 (0.5–0.8)[0.0–1.0]	Proportion of 30-day old calves surviving to 24 months old i.e. deaths between 30 days and 24 months (Number of calves that survived to 24 months/number of calves that survived to 30 days)	The age at which juveniles recruit into the adult population and become sexually mature [29]
**Herd details**			
Females	● 6.6 (5.2–8.3)[2.0–21.0]	Number of females in the herd	Females are the driving force of reproduction in any species, with harem groups recommended for SHO [29]
Herd size	● 8.5 (7.0–10.2)[3.8–23.8]	Number of individuals in the breeding herd	Group size has been found to increase reproductive success in Humboldt penguins [9, 16], flamingos [32] and African wild dogs [33]
Age	● 7.6 (6.9–8.3)[4.6–12.3]	Age of mature individuals in the breeding herd	Reproductive senescence has been shown to affect a wide range of species [20–25]
Transfers	● 4.6 (3.5–5.8)[0.0–15.0]	Total number of previous transfers between zoos per individual	Transfers are rarely tested as a potential source of stress in the literature, but have been shown to negatively affect captive elephants [19]
**Enclosure design**			
Mixed-species	● 0.5 (0.3–0.7)[0.0–1.0]	Keeping of SHO in a mixed-species exhibit (0 = single-species, n = 15; 1 = mixed-species, n = 14)	Replicate naturalistic conditions and are beneficial for zoological institutions, making enclosures more interesting for visitors by housing less charismatic species with more active ones [34]
Stable area	● 97.7 (72.3–126.1)[25.0–311.0]	Land area of the stable (m^2^)	Enclosure size is the most commonly identified variable to influence zoo animal welfare [7–11]
Hardstand area	● 161.2 (89.6–245.5)[0.0–867.0]	Area of the hardstand (m^2^)	
Paddock area	● 20,250 (9650–34,151)[409–150,000]	Area of the paddock (m^2^)	
Outer substrate hardness	● 1.6 (1.4–1.8)[1.0–2.0]	Hardness of the substrate used in the hardstand area (1 = soft, i.e. sand and soil, n = 12; 2 = hard, i.e. concrete, compacted gravel or asphalt, n = 17)	A range of hardstand substrates are used across EEP institutions and some offer only a hardstand, without a grazing paddock [29]
Latitude	● 50.1 (48.6–51.6)[38.0–57.0]	Latitude coordinates of each institution	SHO lived in desert climates, but are often housed in temperate climates [29], thus latitude addresses climatic aspects such as mean annual temperature and rainfall
**Husbandry practices**			
Enrichment	● 0.6 (0.4–0.7)[0.0–1.0]	Provision of enrichment, i.e. browse, branches, brushes or balls (0 = no, n = 13; 1 = yes, n = 16)	Encourages species-specific whilst discouraging stereotypic behaviour, which is vital for the success of reintroduction programmes [35]
Breeding management	● 0.5 (0.3–0.7)[0.0–1.0]	Breeding management strategy (0 = herd together year-round, n = 15; 1 = breeding male separated seasonally, n = 14)	SHO have a strong social structure and hierarchy, thus it can be difficult for separated individuals to reintegrate into the herd [29]
Males present	● 0.5 (0.3–0.7)[0.0–1.0]	Males present during parturition (0 = no, n = 14; 1 = yes, n = 15)	Male presence is not recommended due to resulting winter calves in temperate climates [29]
Post partum public	● 0.6 (0.4–0.7)[0.0–1.0]	Dams visible to public immediately after parturition (0 = no, n = 13; 1 = yes, n = 16)	Females leave the herd to give birth for one week, thus forced close contact with the public may act as a stressor [29]
Annual diet variation	● 0.6 (0.4–0.8)[0.0–1.0]	Diet varied seasonally (0 = no, n = 12; 1 = yes, n = 17)	Some institutions seasonally vary SHO diets in order to maintain winter condition of animals when paddock access is limited and higher energy requirements are needed to cope with thermal stress [29]
Indoor feeding height	● 0.6 (0.4–0.8)[0.0–1.0]	Feeding height in the stable (0 = fed on the floor, n = 11; 1 = food raised above the floor, n = 18)	Floor- and high-level racks can have negative influences on SHO health [29]
Outdoor feeding height	● 0.3 (0.2–0.5)[0.0–1.0]	Feeding height in the outdoor area (0 = fed on the floor, n = 13; 1 = food raised above the floor, n = 16)	
Juvenile physical restraint	● 0.5 (0.3–0.7)[0.0–1.0]	Juveniles (<24 months old) physically restrained (0 = no, n = 14; 1 = yes, n = 15)	Although adults can be trained to move into a crush, physical restraint can cause injuries and horn damage in juveniles [29]
**Public influences**			
Footfall	● 679 (481–932)[10–3,500]	Number of visitors to the institution per year (thousands)	Higher visitor numbers have led to increased vigilance behaviours in other ungulate species [36, 37]
Minimum public distance	● 1.7 (1.2–2.2)[0.0–5.0]	Closest distance the public can get to the SHO (m)	

95% CI = 95% bootstrapped confidence intervals (10,000 iterations).

N = 29 for all variables, with the exception of 30-day and 24-month calf survivorship (N = 27).

All 29 participating institutions were included in our analysis of birth rate, but we excluded two institutions from our analysis of calf survival, which euthanized individuals due to non-breeding recommendations from the EEP coordinator. We also carried out preliminary analysis on the viability of predictor variables. Prior to modelling, predictor variables were reduced to a reliable subset (Table 1) to exclude those with multiple missing observations (n = 9) or low variation (n = 21). To avoid exclusion of further data without bias, for single missing observations in six variables, we inserted the mean value of all other observations [38].

### Data Analysis

Statistical analyses were performed using R (2.14.1; http://cran.r-project.org). Transformations were applied to reduce skew, improve linearity and adjust uneven variances, including log_10_, ln, square root (√) and cube (^3^). For all models, predictor variables were tested for intercorrelation, which can negatively affect the results of regression modelling. Pairs of variables with a Pearson correlation coefficient |r| ≥0.7 were not included in the same model. Therefore, alternative models were run for each breeding response variable to avoid exclusion of potentially important variables [39]. We ran a maximum of four alternative models to avoid overfitting [40]. In addition, all predictor variable sets were checked for Variance Inflation Factors <2 before initialising modelling [39]. Gaussian and binomial GLMs were used to evaluate the influence of predictor variables on birth rate and our two measures of calf survivorship respectively. Predictor variables were also analysed independently for each breeding variable through univariate GLMs, so as to identify any important relationships not recognised by the multivariate models.

Following this, multivariate GLMs were reduced from full models using backward-forward stepwise reduction using the Akaike Information Criterion (AIC), producing a minimum adequate model for each predictor variable data set. GLMs output null and residual deviance values, from which the metric percent deviance explained (%D) can be determined. Similarly to R^2^, %D is a measure of the goodness-of-fit of a model. However, ordinary least squares regression (which outputs R^2^) assumes the response variable has normally distributed errors and is thus based on minimising the squared residual error. GLMs allow for response variables with alternative error distributions and are instead based on maximum likelihood. Accordingly, to modify the regression to match the data type, GLMs must incorporate an error function. %D is calculated by: 1 –(residual deviance/null deviance). Residual deviance will vary according to the error function and %D therefore provides a better model fit when errors are not normally distributed [41].

Finally, to ensure that the reduced models had not suffered from significant reductions in variance, analyses of deviance were applied to each model. Moreover, residual diagnostic plots were created to certify that curvature, heteroscedasticity and leverage (Cook’s D≤1.0) were not having an impact on the modelling process. Furthermore, summary data were calculated as means and bootstrapped confidence intervals (95% CI; 10,000 iterations).

## Results

A total of 275 calves were born to 192 adult SHO females found in the 29 EEP institutions over the period 2010–2014. Mean birth rate over the same period was 1.4 live births female^-1^ (1.2–1.7; n = 29). Mean 30-day calf survivorship was 0.7 (0.6–0.8; n = 27) and mean 24-month calf survivorship was 0.6 (0.5–0.8; n = 27) (Table 1). Enclosure and husbandry predictor variables showed low intercorrelation, with the exception of (1) number of females versus herd size (r = 0.63), (2) paddock area versus stand area (r = 0.58) and stable area (r = 0.79) and (3) herd size versus paddock area (r = 0.70). For birth rate, the three multivariate models identified three predictor variables. Firstly, a positive effect of hardstand substrate hardness was identified in all three of the models where it was included. Positive effects of indoor feeding height and male presence during parturition were also identified (Table 2).

**Table 2:** Predictors of SHO birth rate (N = 29) and 30-day and 24-month calf survivorship (N = 27) from GLMs (2010–2014).

Full model	Minimum adequate model
**Birth rate**	
Mixed-species, √Transfers, Outer substrate hardness, Log_10_ stable area, Log_10_ minimum public distance, Enrichment, Males present, Post partum public, Annual diet variation, Indoor feeding height, Outdoor feeding height	Outer substrate hardness (+), %D = 10.4Males present (+), %D = 8.4%Indoor feeding height (+), %D = 9.1AIC = 63.6, %D = 21.5
Log_10_ herd size, √Transfers, Outer substrate hardness, Log_10_ hardstand area, Log_10_ minimum public distance, Males present, Post partum public, Indoor feeding height	Identical results to the previous model
Ln females, Mean age, Outer substrate hardness, Log_10_ paddock area, Breeding management, Indoor feeding height, Latitude	Outer substrate hardness (+), %D = 6.9, AIC = 64.6
**30-day calf survivorship (cube transformed)**	
Ln females, Mean age, √Transfers, Outer substrate hardness, Log_10_ paddock area, Breeding management, Latitude	Mean age (-), %D = 14.8, AIC = 37.0
Mixed-species, Log_10_ herd size, Mean age, √√Footfall, Males present, Post partum public, Indoor feeding height	Identical result to the previous model.
Mixed-species, Log_10_ herd size, Log_10_ hardstand area, Log_10_ minimum public distance, Males present, Juvenile physical restraint	Log herd size (+), %D = 10.3, AIC = 36.3
**24-month calf survivorship**	
Log_10_ herd size, Outer substrate hardness, Log_10_ stable area, Log_10_ minimum public distance, Enrichment, Juvenile physical restraint, Latitude	Outer substrate hardness (+), %D = 15.6, AIC = 34.8
Ln females, Mean age, Log_10_ paddock area, √Transfers, Breeding management, Annual diet variation, Indoor feeding height	Mean age (-), %D = 19.6, AIC = 35.0
Mixed-species, Outer substrate hardness, Log_10_ hardstand area, Enrichment, Indoor feeding height, Outdoor feeding height, Juvenile physical restraint	Outer substrate hardness (+), %D = 27.9Indoor feeding height (+), %D = 14.0Outdoor feeding height (+), %D = 8.7AIC = 34.0, %D = 35.7
Mixed-species, Outer substrate hardness, √√Footfall, Enrichment, Indoor feeding height, Outdoor feeding height	Identical result to the previous model.

• √ = square root, Log = log_10_ and Ln = natural log.

• The direction of the trend (+/-) and percent deviance explained (%D) are included.

• Minimum adequate models did not show reduced deviance from full models (Analysis of Deviance: p = 0.62–0.98).

• An additional alternative model for 30-day calf survivorship did not converge: mixed-species, log_10_ herd size, log_10_ stable area, enrichment, post partum public, annual diet variation, indoor feeding height, outdoor feeding height and juvenile physical restraint.

**Fig 1:**
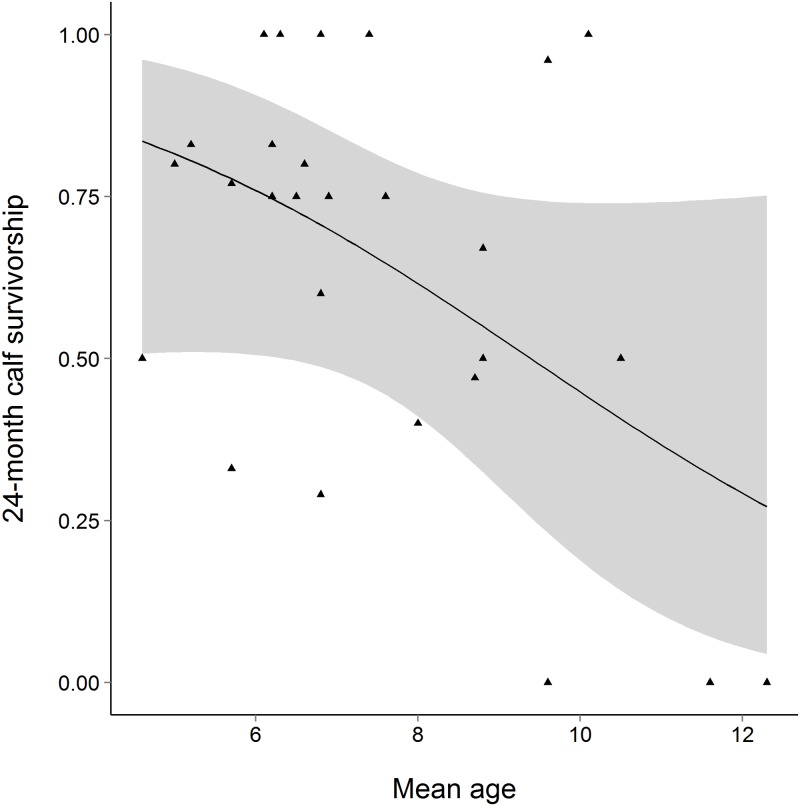
The effect of the mean age of breeding individuals on SHO calf survivorship. The effect of the mean age of mature individuals in the breeding herd (years) on 24-month calf survivorship. The solid line represents the univariate logistic regression line. Shaded regions indicate binomial 95% confidence intervals around the regression line.

**Fig 2:**
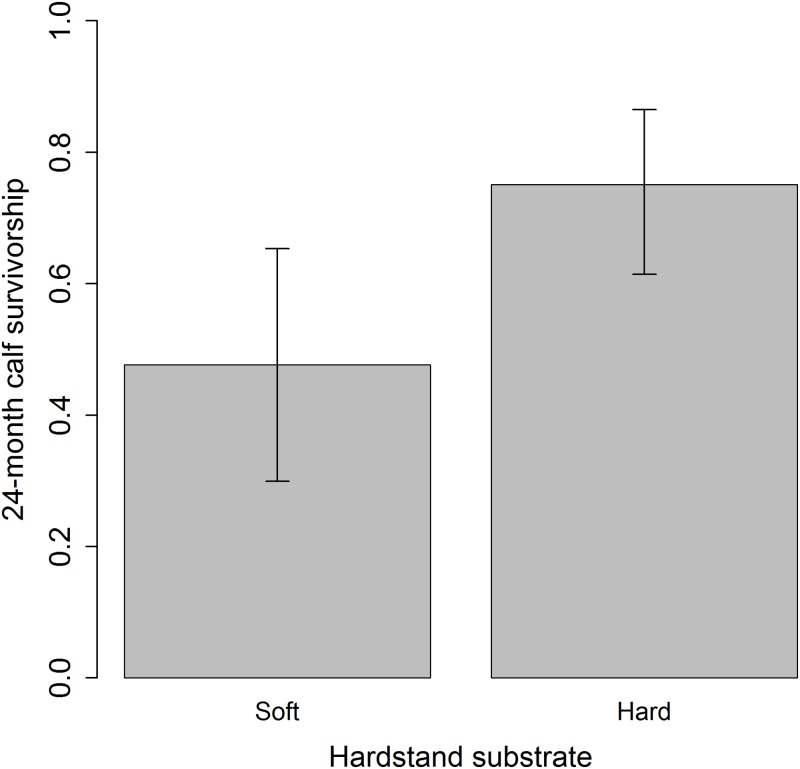
Variation in 24-month calf survivorship with differing hardstand substrates. Sand and soil are defined as “Soft” substrates; concrete, asphalt and compacted gravel are defined as “Hard” substrates. Bars represent means ± 95% bootstrapped confidence intervals.

Three multivariate models for 30-day calf survivorship identified two predictor variables. Two separate models found that increasing mean age of mature individuals negatively impacted 30-day calf survivorship. A positive effect of increasing herd size was also found in one of the two models where it was included (Table 2). Additionally, four multivariate models for 24-month calf survivorship identified four predictor variables. Negative and positive effects of mean age of mature individuals and hardstand substrate hardness respectively were found for 24-month calf survivorship (Table 2; Figs 1 and 2). Hardstand substrate hardness was identified in all three of the models where it was included, explaining up to 27.9% of the deviance (Table 2). Indoor and outdoor feeding height also had positive impacts (Table 2).

Across all three measures of breeding success, 47 univariate models for variables that did not appear in minimum adequate models did not identify any additional predictor variables with greater influence on breeding (%D = 0.006–7.2; AIC = 37.5–66.6). A single exception was increasing 24-month calf survivorship with breeding management through male separation from the rest of the herd (%D = 10.3, AIC = 38.2). Finally, we ran an additional univariate model to determine if the negligible effects of latitude were due to zoos in temperate climates removing males post-partum. Indeed, this was found to be the case (%D = 28.7, AIC = 38.25).

## Discussion

Our results suggest that there are a minimal number of enclosure design and husbandry variables having an effect on SHO breeding success. However, over one third of the variation in 24-month calf survivorship is explained by the few enclosure design and husbandry variables that are having an effect, thus it is clear that some improvements to the guidelines are necessary if conservation breeding is to be optimal. We here discuss the implications for zoo management and conservation science.

Whilst the influence of the hardstand substrate was found for both birth rate and 24-month calf survivorship, its influence on the latter explained the most variance in SHO breeding success. The abrasiveness of hard substrates helps to prevent overgrown hooves, which can cause pain, reduce activity and affect competitive behaviour [42], presumably including mating. SHO are also intolerant of wet conditions, which can cause foot-rot [43]. Harder substrates are easier to clean and offer more efficient drainage, thus improving hygiene conditions and reducing parasite intensity. Indeed, parasitic infections have been associated with stillbirths in another antelope species [44]. Whilst the husbandry guidelines suggest that harder substrates improve hygiene conditions, they still state that a range of hardstand substrates can be used [29], thus the use of harder substrates clearly needs to be better enforced.

Our observed relationship between mean herd age and both 30-day and 24-month calf survivorship suggests that ageing herds have reduced breeding success. Specifically, our data suggest that 24-month calf survivorship is lowest for herds with a mean age of breeding above 10 years (Fig 1). This general trend concurs with studies on a wide range of species (red wolves [20]; tigers [21]; red-billed choughs [24]; western lowland gorillas [25]), including ungulates (European roe deer [22]; reindeer [23]). Presumably, this relates to decreasing reproductive quality of ageing individuals (reproductive senescence) [45]. The husbandry guidelines do not currently state a suitable mean age for a breeding herd. We therefore propose more specific recommendations, i.e. that reproduction should be prioritised from female SHO below the age of 10 years.

Weaker positive influences of feeding height for birth rate and 24-month calf survivorship and mean herd size for 30-day calf survivorship, also suggest further considerations for management. Previous studies have found that using platforms and high positioning of troughs reduces agonistic interactions in goats [46] and horses [47]. Food competition can negatively influence lower-ranking individuals in ungulate species, as it results in them achieving a lower calorie intake [46]. Indoor feeding height thus appears to have a greater impact than outdoor feeding height, as the opportunity for paddock grazing may reduce conflict over additional food resources outside. Furthermore, the husbandry guidelines indicate that placing racks on the floor can cause injury from panic behaviour. However, they also outline that hay can be placed on the floor and that racks should not be placed too high due to respiratory problems resulting from dust inhalation [29]. As a result, we propose that the husbandry guidelines should promote the raising of the indoor feeding height, i.e. using racks/troughs rather than hay floor piles or pellet bowls, but without the racks being above head height.

The husbandry guidelines also indicate that before their extinction in the wild, SHO lived in herd sizes of approximately 10–30 animals [29], yet the mean herd size of the institutions included in our study is just 8.5. Nevertheless, the ability of institutions to increase herd sizes will of course be influenced by financial and space constraints. In addition, more-specific husbandry guidelines are easier to instigate in smaller herds, thus many institutions are in the process of reducing herd sizes. Furthermore, these wild populations consisted of equal sex ratios, which is not possible to re-create in zoological institutions due to intra-specific aggression. Thus any increases in group size would involve an increase in the number of females only. This would have potentially conflicting effects on population management given that any increases in the ratio of females to males would impact on the retention of genetic diversity [48]. Hence whilst larger herd sizes may increase breeding success, they could have negative influences on future reintroduction plans for the species and so we are reluctant to suggest specific herd sizes here.

Our observed increase in birth rate when males are present during parturition is likely a result of increased mating opportunity, rather than an underlying factor such as separation stress. Females can re-conceive during their post-partum oestrus [29], thus if males are present during this period, this will result in a greater number of pregnancies. Furthermore, in Northern European zoos, allowing females to breed during their post-partum oestrus results in winter births, when SHO are usually restricted to the stable and hardstand areas [29]. This results in abnormally high mother-calf contact and hence overfeeding, which results in *E*. *coli* infections and thus calf mortality [29]. Therefore, winter births resulting from continual male presence are also likely to result in decreased overall survivorship, hence explaining our observed increase in 24-month calf survivorship with male separation. Consequently, in agreement with the current husbandry guidelines, we would not recommend male presence during parturition.

The captive environment typically presents environmental constraints and close human proximity that place added pressure on captive individuals compared to their wild counterparts [49]. However, besides those variables discussed, several enclosure and husbandry features were not found to influence SHO breeding success. Most encouraging for achieving conservation breeding in a human environment are negligible influences from annual footfall and minimum public distance. The observed lack of influence of mixed-species exhibits is also positive, as these are common for SHO (56% of institutions, n = 22) and are useful to zoological institutions through reducing costs and increasing educational value [34]. Furthermore, the negligible influence of climatic aspects in our models clearly indicates that institutions in temperate environments are capable of successful breeding, providing they remove males post-partum.

Of the two most influential variables for SHO breeding success, only one is commonly reported in the literature. As aforementioned, the negative effect of age is widespread across multiple species. However, it is surprising that the effect of enclosure substrates is not widespread throughout the literature, especially as the use of the hardstand substrate explained the most variance in breeding success in our study. Notwithstanding, the effect of enclosure substrates on breeding success is rarely tested, with studies which do incorporate them being biased towards birds (Humboldt penguins [16]; flamingos [50]). Future enclosure design studies on other taxa may therefore benefit from incorporating substrate types.

Whilst our results indicate that the number of enclosure/husbandry influences on breeding are relatively few compared to previous studies, due to the multi-institutional questionnaire approach, this study addresses herd-level data only. Investigating the effects of individual level data such as calf sex [51], and degree of inbreeding/genetic variation [52, 53] would strongly benefit herd management. Secondly, our findings would benefit from future experimental investigation of the key variables to determine causality rather than correlation. Certainly, this would aid in the justification of our revised guideline suggestions. Of course, developing fully replicated experimental trials for the 20 enclosure and husbandry variables included here would be unlikely to happen. However, given that our results suggest only six variables are influencing the breeding success of the captive population, the cost and impracticality of experimental manipulation has been substantially reduced. Future work should therefore aim to experimentally determine if these six variables indeed have the predicted beneficial effects on breeding success.

Despite the potential for further work, with only two variables explaining >19% deviance in breeding, it is clear that enclosure design and husbandry are having less influence on SHO than other tested species. In all papers cited here using regression analyses (n = 8), three to eight variables per paper were found to explain 19% to 83% deviation in breeding success [9, 10, 16, 18, 22, 23, 32, 54]. Nevertheless, a sustainable insurance population relies on optimising breeding success. Previous studies have used benchmarks from wild/working populations to assess the success of conservation breeding [55], suggesting that infant losses in zoos should be no greater than 10% for captive elephants [51]. Currently, there is not enough statistically viable SHO benchmark data for comparison. However, calf mortality rates in the institutions included here range from 20–50% (Table 1), hence some institutions are clearly performing better than others. Accordingly, our data suggest some potential improvements and hence that extinct-in-the-wild species stand a greater chance of survival with empirical design of zoo enclosures and husbandry methods.

## Supporting Information

S1 Appendix Questionnaire on SHO management sent to all SHO EEP institutions. (DOCX)
