# Supplementary material for: Evaluating Conservation Breeding Success for an Extinct-in-the-Wild Antelope
Source: PLoS One. 2016 Dec 9;11(12):e0166912. doi: 10.1371/journal.pone.0166912 (PMC5147836; doi:10.1371/journal.pone.0166912)
Supplement: S1 Appendix — (DOCX) [file pone.0166912.s001.docx]

**S1 Appendix. Questionnaire on SHO management sent to all SHO EEP institutions.**

**Enclosure design**

Are your SHO in a mixed-species exhibit?

Yes No

If yes, please tick all that apply:

Rhinoceros

Grevy’s zebra

Hartmann’s mountain zebra

Ostrich

Giraffe

Lechwe

Gazelle (please specify species)

Other (please specify)

If yes, please provide the number of individuals of each species that currently share the enclosure:

Do you have a diagram of your enclosure with specific measurements for the stable, hardstand and paddock areas?

Yes No

If yes please provide, if no would you be willing for me to come and collect this information?

Yes No

What substrate is the major substrate inside the stable?

Concrete Asphalt Rubber

Other (please specify):

What substrate do you provide for bedding (tick all that apply)?

Sawdust Bark Straw

Other (please specify):

What is the major substrate type in your hardstand area outside?

Concrete Compacted gravel Sand

Other (please specify):

What type of barriers surround the perimeter of your enclosure (tick all that apply)?

Fence Hedge Ditch

Other (please specify)

At the closest point how close can members of the public get to the SHO?

What proportion of the perimeter of the enclosure do members of the public have access to?

What is the estimated annual footfall in the zoo? If the footfall is known for the specific area that the enclosure is in please provide this too.

Do you provide enrichment in the enclosure? This includes browse and other food-related enrichment.

Yes No

If yes, how often do you provide enrichment?

Daily Weekly Fortnightly Monthly Constant Other

If yes, please specify type:

Do your keepers go into the enclosure with the SHO?

Yes No

If yes, do they enter enclosures with pregnant females?

Yes No

If yes, do they enter enclosures with neo-natal (<30 days old) calves?

Yes No

If yes, do they enter enclosures with calves (>30 days old) and juveniles?

Yes No

Are there dedicated SHO keepers (yes), or do keepers rotate (no)?

Yes No

Has your SHO enclosure changed in the last 3 years? If yes, please specify when and how:

**Herd management**

Are your SHO held in more than one group? If yes how many groups (this does **not** include separation for breeding management)?

Yes How many:

No

**Breeding management**

Do you employ a breeding strategy that involves separating individuals e.g. the breeding male, from the herd at a certain time of year?

Yes No

If yes, in what month do you separate them from the herd? Please specify number of individuals and gender.

If yes, in what month do you reintroduce them to the herd? Please specify number of individuals and gender.

If yes, during separation do they have access to the following? (tick all that apply)

Stable Hardstand area Grazing paddock

During separation, how many months do they have access to a grazing paddock?

If separated, what is the distance of the separation area from the area accessed by the remaining herd?

If separated, is the separation area the same size, smaller or larger than the area accessed by the rest of the herd?

Stable Same Smaller Larger

Hardstand area Same Smaller Larger

Grazing paddock Same Smaller Larger

If smaller or larger, do you have a diagram with measurements of the separation area(s)?

Yes No

If yes, please provide, if no, would you be willing for us to come and collect this information?

Yes No

At what age do you separate juveniles from the herd?

Are pregnant females separated from the herd prior to parturition?

Yes No

Are new dams and calves separated from the herd post-partum?

Yes No

If yes, why?

If yes, when are they reintroduced to the herd?

Are adult males present in the herd during parturition?

Yes No

Are the public able to view dams during parturition?

Yes No

After what period of time are the public able to view dams post-partum?

Are dams separated from calves during the 30 days post-partum?

Yes No

If yes, why?

Are calves captured <30 days post-partum?

Yes No

If yes, why?

**Diet and nutrition**

Please provide a breakdown of diet and the proportions for each component or attach a diet sheet.

Does the diet provided vary over a year?

Yes No

If yes, please provide diet sheets for different seasons.

Do you feed the animals indoors?

Yes No

If yes, please tick method and specify how many.

Racks How many:

Troughs How many:

Floor

Other (please specify):

Do you feed the animals outdoors?

Yes No

If yes, please tick method and specify how many.

Racks How many:

Troughs How many:

Floor

Grazing only

Other (please specify):

**Transport**

How do you transport SHO? (tick all that apply)

Crate Loose Box

Other (please specify):

**Restraint**

How do you restrain SHO? (tick all that apply)

Physical restraint

Yes No

Chemical restraint

Yes No

If physical restraint, please specify method for adults:

Crush

Human restraint only

Drop floor chute/tamer

Other (please specify):

If physical restraint, please specify method for juveniles:

Crush

Human restraint only

Drop floor chute

Other (please specify):

**Health**

What is the general body condition of your herd(s) of SHO?

Fat Normal Thin Emaciated

Do you weigh your oryx?

Yes No

If yes, do you use:

Scales built into the enclosure Moveable scales

If yes, please provide individual weights for the oryx:

How often do individuals suffer physical injuries that have to be treated (including disinfectant spray)?

Daily Weekly Monthly Yearly Random (no pattern)

Other (please specify):

If individuals suffer physical injuries what are their causes? (tick all that apply)

Self-inflicted Conspecifics Objects in the enclosure

Other species in a mixed-exhibit (please specify species) Other (please specify):

Would you be willing to provide ZIMS, ARKS, MedARKS and post-mortem reports to the EEP co-ordinator for all current individual SHO in your collection, for confidential use in this study? If yes, please provide them.

**Previous studies**

Have any behavioural/space use/visitor effect studies been carried out on your collection?

Yes No

If yes, please provide if willing.

These questionnaires were sent to 60 institutions. These institutions were chosen as they are current members of the EEP (European Endangered species Programme) and thus are actively involved in conservation breeding efforts for scimitar-horned oryx. The following institutions (who wish to be acknowledged) are those we received replies from and thus participated in our study (Aalborg Zoo, African Safari, Artis Royal Zoo, Attica Park, Berlin Zoo, Zoo de la Boissiere du Doré, Cerza Zoo, Chester Zoo, Parco Faunistico le Cornelle, Cotswold Wildlife Park and Gardens, DierenPark Amersfoort, Dublin Zoo, Flamingo Land Resort, Fota Wildlife Park, Parco Natura Viva – Garda Zoological Park, Miejski Ogród Zoologiczny Wybrzeza w Gdansku (Zoo Gdansk), Jérusalem Zoo, Parc Zoologique de Jurques, Karlsruhe Zoo, Knowsley Safari Park, Zoo Dvur Kralove, Zoo Krefeld, Marwell Zoo, Monde Sauvage Safari Parc, Parc Darwin – Parc Zoologique de Montpellier, Le Pal, Zoo la Palmyre, Wild Animal Park Planckendael, Planète Sauvage, Plock Zoo, Zoological Garden Prague, Zoological Center Tel Aviv – Ramat Gan, Parc Zoologique de Thoiry, Réserve de la Haute Touche, Woburn Safari Park, Zoo Wroclaw LLd., Zoological Garden of Zagreb and ZSL Whipsnade Zoo).
